# Differential organization of cortical inputs to striatal projection neurons of the matrix compartment in rats

**DOI:** 10.3389/fnsys.2015.00051

**Published:** 2015-04-14

**Authors:** Yunping Deng, Jose Lanciego, Lydia Kerkerian-Le Goff, Patrice Coulon, Pascal Salin, Philippe Kachidian, Wanlong Lei, Nobel Del Mar, Anton Reiner

**Affiliations:** ^1^Department of Anatomy and Neurobiology, The University of Tennessee Health Science CenterMemphis, TN, USA; ^2^Neurosciences Division, Center for Applied Medical Research (CIMA), Centro de Investigación Biomédica en Red sobre Enfermedades Neurosdegenerativas (CIBERNED), and Instituto de Investigación Sanitaria de Navarra (IdiSNA), University of Navarra Medical CollegePamplona, Spain; ^3^Aix-Marseille Université, CNRS, IBDM UMR 7288Marseille, France; ^4^Aix Marseille Université, CNRS, INT UMR 7289Marseille, France; ^5^Department of Anatomy, Zhongshan Medical School of Sun Yat-Sen UniversityGuangzhou, China

**Keywords:** basal ganglia, corticostriatal, thalamostriatal, projection neurons, axospinous, axodendritic

## Abstract

In prior studies, we described the differential organization of corticostriatal and thalamostriatal inputs to the spines of direct pathway (dSPNs) and indirect pathway striatal projection neurons (iSPNs) of the matrix compartment. In the present electron microscopic (EM) analysis, we have refined understanding of the relative amounts of cortical axospinous vs. axodendritic input to the two types of SPNs. Of note, we found that individual dSPNs receive about twice as many axospinous synaptic terminals from IT-type (intratelencephalically projecting) cortical neurons as they do from PT-type (pyramidal tract projecting) cortical neurons. We also found that PT-type axospinous synaptic terminals were about 1.5 times as common on individual iSPNs as IT-type axospinous synaptic terminals. Overall, a higher percentage of IT-type terminals contacted dSPN than iSPN spines, while a higher percentage of PT-type terminals contacted iSPN than dSPN spines. Notably, IT-type axospinous synaptic terminals were significantly larger on iSPN spines than on dSPN spines. By contrast to axospinous input, the axodendritic PT-type input to dSPNs was more substantial than that to iSPNs, and the axodendritic IT-type input appeared to be meager and comparable for both SPN types. The prominent axodendritic PT-type input to dSPNs may accentuate their PT-type responsiveness, and the large size of axospinous IT-type terminals on iSPNs may accentuate their IT-type responsiveness. Using transneuronal labeling with rabies virus to selectively label the cortical neurons with direct input to the dSPNs projecting to the substantia nigra pars reticulata, we found that the input predominantly arose from neurons in the upper layers of motor cortices, in which IT-type perikarya predominate. The differential cortical input to SPNs is likely to play key roles in motor control and motor learning.

## Introduction

The dendritic spine-rich striatal projection neurons (SPNs) are organized into at least three major types, direct pathway striatal projection neurons (dSPNs) of the matrix compartment, indirect pathway striatal projection neurons (iSPNs) of the matrix compartment, and projection neurons of the patch (or striosomal) compartment (Gerfen, 1984, 1989, 1992; Graybiel, 1990; Kawaguchi et al., 1990; Reiner and Anderson, 1990; Gangarossa et al., 2013). These SPN types differ in their neurochemistry, projection targets and roles in basal ganglia function. The dSPNs are enriched in substance P (SP), dynorphin, and D1 type dopamine receptors, project primarily to the internal pallidal segment (GPi) and/or the substantia nigra pars reticulata (SNr), but with collaterals to the external pallidal segment (GPe), and they are widely thought to play an important role in movement initiation (Albin et al., 1989; DeLong, 1990; Reiner and Anderson, 1990; Kravitz et al., 2010). The iSPNs are enriched in enkephalin and D2 type dopamine receptors, project exclusively to the GPe, and play a role in suppression of unwanted movements (Albin et al., 1989; Sano et al., 2003; Kravitz et al., 2010; Cui et al., 2013; Freeze et al., 2013). Striosomal projection neurons are enriched in SP, dynorphin, and/or enkephalin, mu opiate receptors, and D1 and/or D2 dopamine receptors, project to dopaminergic neurons of the substantia nigra pars compacta (SNc), and thereby modulate the dopaminergic influence on striatum (Graybiel, 1990; Gerfen, 1992; Wang et al., 2007; Perreault et al., 2010, 2011).

In prior studies in rats, we have found that dSPNs and iSPNs differ in the cortical neuron types from which they receive the majority of the input to their spines (Lei et al., 2004; Reiner et al., 2010). In brief, two major cortical neuron types project to striatum, the neuron types referred to as the intratelencephalically projecting type (IT-type) preferentially localized to upper layer 5 and the pyramidal tract type (PT-type) preferentially localized to lower layer 5 (Wilson, 1987; Cowan and Wilson, 1994; Reiner et al., 2003). The IT-type neurons appear to send a motor planning and sensory signal to striatum, while PT-type neurons send an efference copy signal of motor commands to striatum (Turner and DeLong, 2000; Reiner et al., 2010). Our previous EM studies have indicated that dSPNs receive the majority of their cortical axospinous input from IT-type cortical neurons (Lei et al., 2004; Reiner et al., 2010). This accords well with the role of this SPN type in movement initiation and with the motor planning signal conveyed by the IT-type input. By contrast, we have found that iSPNs receive the majority of their axospinous input from PT-type corticostriatal neurons, which may be important for a role in motor sequence termination. Our prior studies, however, have not resolved the relative amounts of IT-type vs. PT-type input to dSPNs vs. iSPNs, nor have they ascertained whether the SPN types differ in the types of cortical neurons from which they receive their input on dendrites.

Several morphological and electrophysiological studies have reported findings consistent with ours in rodents and primates (Uhl et al., 1988; Berretta et al., 1997; Parthasarathy and Graybiel, 1997; Mallet et al., 2006; Cepeda et al., 2008; Ding et al., 2008; Kreitzer and Malenka, 2008; Inoue et al., 2012; Takada et al., 2013), although others have not (Ballion et al., 2008; Kress et al., 2013; Wall et al., 2013). Among the latter, one optogenetic study reported a stronger PT-type response in dSPNs than iSPNs, and equal IT-type responses (Kress et al., 2013), while a study using transneuronal viral tracing reported similar numbers of upper vs. lower layer 5 cortical neurons projecting to both SPN types (Wall et al., 2013). The viral tracing study of Wall et al. (2013) also reported dSPNs as receiving greater cortical input from limbic and sensory rather than motor regions. In the present study, we have used EM double-label methods to examine in more detail the differential sizes and abundance of IT-type and PT-type inputs to the spines and dendrites of dSPNs and iSPNs in rats. We have also used rabies virus retrograde transneuronal labeling to examine the source of cortical input to dSPNs projecting to SNr. Our studies show that cortical input to dSPNs in rats arises primarily from upper layer 5 neurons of the primary and secondary motor cortices (presumptively mainly IT-type), with the overall IT-type input to dSPN spines being about twice as common as the overall PT-type input to dSPN spines. For iSPNs, PT-type input to spines appears to be about 1.5-fold more common than IT-type input is to spines. Notably, the IT-type terminals on iSPN spines are significantly larger than those on dSPN spines. The axodendritic IT-type terminals appear to be largely comparable for the two SPN types in their size and abundance, but the axodendritic PT-type terminals appear to be more abundant on dSPNs than iSPNs, although no different in size. Our findings may help explain the basis of some of the discordant results obtained by others, as detailed in the Discussion.

## Materials and Methods

### EM Studies in Rats of Corticostriatal Terminals on SPNs

#### Subjects

EM results from 14 adult male Sprague Dawley rats (Harlan, Indianapolis, IN) are presented here. These were previously used in Lei et al. (2004), and were subjected here to detailed analysis not performed in the prior study. All animal use was performed in accordance with the National Institutes of Health *Guide for Care and Use of Laboratory Animals*, Society for Neuroscience Guidelines, and University of Tennessee Health Science Center Guidelines.

#### BDA Injections and Immunolabeling

We sought to characterize the size and relative abundance of the IT-type and PT-type terminals making contact with spines vs. dendrites enriched in D1 dopamine receptors, thereby focusing on dSPNs, vs. those ending of D1− spines and dendrites, thereby focusing on iSPNs (Gerfen, 1992; Le Moine and Bloch, 1995). This allowed us to make comparisons for the two SPN types in the same cases. Included among the neurons immunolabeled for D1 are those projecting primarily to the nigra, as well as those projecting both to GPi and nigra (Kawaguchi et al., 1990; Hersch et al., 1995; Parent et al., 1995; Wu et al., 2000). Note that use of D1− immunolabeling to detect iSPNs has been employed by others as well (Day et al., 2006; Lei et al., 2013; Deng et al., 2014). Although use of D1− immunolabeling runs the risk of identifying dSPN spines and dendrites that have failed to immunolabel for D1 as iSPNs, this risk is minimized in our tissue because we only performed analysis in fields in which the abundance of D1+ spines and D1− spines was comparable (across all cases: 408 D1+ spines, 420 D1− spines). Note that use of D2 immunolabeling for detecting iSPNs is more problematic, because about 40% of dSPNs possess D2 (Deng et al., 2006).

The effectiveness of our approach for analyzing cortical input to SPN dendrites requires that we address the possibility that some D1+ and/or D1− dendrites might belong to striatal interneurons. For D1+ dendrites, this concern is minimal. Available published data indicate that D1 receptors are scarce or absent on somatostatinergic interneurons (which also typically contain nNOS and NPY), cholinergic interneurons, parvalbuminergic interneurons, and calretinergic interneurons (Dawson et al., 1990; Le Moine et al., 1991; Centonze et al., 2003; Petryszyn et al., 2014). Moreover, somatostatinergic and cholinergic striatal interneurons receive scant cortical input (Dimova et al., 1993; Tepper et al., 2010). Thus, those IT-type and PT-type terminals contacting D1+ dendrites are extremely likely to be contacting dSPN dendrites. Additionally, since cholinergic and somatostatinergic interneurons receive little cortical input (Tepper et al., 2010), D1−negative dendrites receiving IT-type or PT-type terminals are also unlikely to belong to cholinergic and somatostatinergic interneurons. Parvalbuminergic interneurons do, however, clearly receive cortical input, and calretinergic interneurons may as well (Tepper et al., 2010). Although these two neuron types together only account for about 2% of all striatal neurons and their dendrites are thus far less abundant than those of iSPNs, it is possible that some of D1− dendrites with IT-type or PT-type terminals belonged to these interneuron types. This possibility is taken into consideration in our interpretations and conclusions.

Our methods for selective BDA labeling of IT-type and PT-type terminals have been described in detail and illustrated (Reiner et al., 2000, 2003; Reiner, 2010), and are summarized here. For studies of IT-type terminals, six rats received unilateral injections of BDA10k (dextran, biotinylated, 10,000 MW, anionic, lysine fixable; Molecular Probes) into motor cortex on the left side of the brain. In these cases, anterograde corticostriatal labeling in the right striatum would be limited to IT-type terminals, because PT-type corticostriatal neurons do not project to contralateral striatum, but IT-type neurons do (Cowan and Wilson, 1994; Reiner et al., 2003). Before surgery, animals were deeply anesthetized with ketamine (87 mg/kg) and xylazine (13 mg/kg). A 1 µl Hamilton microsyringe was used to inject 0.1–0.2 µl of 5% BDA10k in 0.1 M sodium phosphate buffer (PB), pH 7.4, using stereotaxic methods as described previously (Reiner et al., 2003). For studies of PT-type terminals, 0.15 µl of 10% BDA3k (dextran, biotinylated, 3000 MW, anionic, lysine fixable; Molecular Probes) in 0.1 M sodium citrate-HCl, pH 3.0 (Reiner et al., 2000) was injected into the pyramidal tract at pontine levels in 8 rats, using a 1 µl Hamilton microsyringe, which produces selective labeling of the intrastriatal collaterals of PT-type neurons (Reiner et al., 2003). Sections were processed for D1 immunolabeling, and all were processed for ABC visualization of BDA labeling in corticostriatal terminals.

#### Tissue Fixation and Processing

After 7–10 days, the rats that had been injected with BDA were deeply anesthetized with 0.8 ml of 35% chloral hydrate in saline and then perfused transcardially (Lei et al., 2004). The rats were first exsanguinated by perfusion with 30–50 ml of 6% dextran in PB, followed by 400 ml of 3.5% paraformaldehyde– 0.6% glutaraldehyde–15% saturated picric acid in PB, pH 7.4. Brains were removed, postfixed overnight in the same fixative without glutaraldehyde, and then sectioned at 50 µm on a vibratome. Tissue was processed first by the ABC procedure for BDA localization and then immunolabeled for D1. The sections were pretreated with 1% sodium borohydride in 0.1 M PB for 30 min followed by incubation in 0.3% H_2_O_2_ solution in 0.1 M PB for 30 min. BDA was then visualized by using the ABC Elite kit (Vector Laboratories), using a nickel-intensified DAB procedure as described previously (Reiner et al., 2003). These sections were subsequently washed six times in PB, and immunohistochemical labeling for D1 was performed using a brown DAB reaction. After BDA visualization of corticostriatal terminals and D1 immunolabeling of spines, the sections were rinsed in 0.1 M sodium cacodylate buffer, pH 7.2, postfixed for 1 h in 2% osmium tetroxide (OsO_4_) in 0.1 M sodium cacodylate buffer, dehydrated in a graded series of ethyl alcohols, impregnated with 1% uranyl acetate in 100% alcohol, and flat embedded in Spurr’s resin (Electron Microscopy Sciences, Fort Washington, PA). For the flat embedding, the sections were mounted on microslides pretreated with liquid releasing factor (Electron Microscopy Sciences). The Spurr’s resin-embedded sections were examined light microscopically for the presence of BDA-labeled axons and terminals in striatum. Pieces of embedded tissue were then cut from the dorsolateral striatum and glued to carrier blocks, and ultrathin sections were cut from these specimens with a Reichert ultramicrotome. Since striosomes are sparse in dorsolateral striatum (Wang et al., 2007), our findings are focused primarily on matrix SPNs. The sections were mounted on mesh grids, stained with 0.4% lead citrate and 4.0% uranyl acetate using an LKB-Wallac (Gaithersburg, MD) Ultrastainer, and finally viewed and photographed with a Jeol (Peabody, MA) 1200 electron microscope.

Analysis and quantification was performed using the images of the EM labeling. To analyze the material, we located the BDA-labeled terminals that made asymmetric synaptic contact with spine heads or dendrites in the striatum. Synaptic contacts were identified by the presence of synaptic vesicles in the terminal and a postsynaptic density in the target. At the EM level, the black nickel-intensified DAB reaction product resembles the brown DAB reaction in its diffuse and flocculent appearance. The similarity in appearance of the nickel-intensified and standard brown DAB reaction products at the EM level, however, was not a hindrance because corticostriatal terminals and dendritic spines of striatal neurons are morphologically distinct structures. Moreover, because BDA-labeled corticostriatal terminals were intensely labeled with DAB, they could be distinguished from the rare D1+ immunolabeling of excitatory axospinous synaptic terminals (3.1% of all asymmetric axospinous synaptic terminals), which tend to be only lightly labeled (Hersch et al., 1995; Lei et al., 2004). Finally, because spiny neurons do not project to pons or cortex, BDA injections into either site do not yield labeling of striatal neurons (Reiner and Anderson, 1990). Thus, our approach did not yield BDA+ spines that might be confused with D1 immunolabeled spines. The overall EM analysis presented is based on over 800 axospinous and axodendritic synaptic terminals. Of these, about 20% were labeled for BDA. We tabulated the size of terminals and type of structures contacted. Terminals were measured at their widest diameter parallel to and 0.1 µm from the postsynaptic density.

### Rabies Virus Transneuronal Retrograde Labeling in Rats

Five male Wistar rats with a body weight ranging from 240–280 g were used. These animals were used in a prior study on transneuronal striatal interneuron labeling from the substantia nigra (Salin et al., 2009). The animals were handled according to European Council Directive 86/609/EEC and all experimental procedures were approved by the Ethical Committee for Animal Testing of the University of Navarra (Ref: 010-06). The use of rabies virus was carried out in a biosafety level 2 laboratory and all the personnel involved had been previously vaccinated. The viral strain used was Challenge Virus Standard (CVS-11), which is commonly used in transneuronal tracing experiments, particularly when injected centrally (Kelly and Strick, 2000). The animals were anesthetized with an intraperitoneal injection of equithesin (4 mL/kg) and placed in a stereotaxic frame (David Kopf, Tujunga, CA). Subsequently, rabies virus was pressure-injected as a cell culture supernatant in a final volume of 300 nL in minimal essential medium, titrated at 4 × 10^7^ plaque forming units/mL, at coordinates targeting the substantia nigra, pars reticulata. As reported previously (Salin et al., 2008, 2009), the post-injection survival time was adjusted to detect first-order infected SPNs, and to limit second-order infection to those regions having input to the first order neurons having the greatest input to SNr. Since striatonigral dSPNs have far and away the greatest input to SNr, and accordingly contain the greatest number labeled by primary retrograde labeling, the labeled neurons in cerebral cortex are largely those projecting directly to striatonigral dSPNs. For the cases presented here, the injections were limited to the SNr, as illustrated previously (Salin et al., 2009). Several other brain regions in addition to striatum project to SNr and themselves receive cortical input (Naito and Kita, 1994a; Gerfen and Bolam, 2010). These could in principle be additional routes by which CVS-11 rabies virus (RV) injected into SNr could yield cortical neuronal labeling. These regions include GPe and subthalamic nucleus (STN). Few labeled neurons were, however, observed in GPe or STN after RV injection in SNr (typically no more than a 5−10 per section) compared to the large number of infected striatal dSPNs in any given section (hundreds in the regions of highest abundance). The paucity of GPe labeling is consistent with the restricted nature of our injections, which were limited to SNr and did not include SNc, to which GPe more heavily projects (Reiner et al., 1998; Gerfen and Bolam, 2010). Hypothalamic regions also project to substantia nigra (Fallon et al., 1985; Peyron et al., 1998; Lee et al., 2008), and could be another source of cortical labeling with RV injection into SNr. Hypothalamic projections, however, more heavily target SNc than SNr, and we saw no RV+ neurons in hypothalamus. Finally, although cerebral cortex projects to SNc, it projects only lightly to SNr (Naito and Kita, 1994b), which argues against the likelihood of direct cortical labeling from our SNr injections.

After a survival time of 40–42 h post-viral delivery, the animals were anesthetized with an overdose of equithesin and perfused transcardially with a saline solution followed by 500 mL of a fixative solution containing 4% paraformaldehyde in 0.125 M PB (pH 7.4). After perfusion, the brain was removed and stored in a cryoprotectant solution containing 20% glycerin and 2% dimethylsulphoxide in 0.125 M PB (pH 7.4). Frozen coronal sections (40 µm thick) were collected in 0.125 M PB (pH 7.4) in 10 series of adjacent sections. One series was used for immunofluorescent detection of rabies virus. A detailed description of the procedure for visualization of rabies virus is given elsewhere (Salin et al., 2008, 2009). In brief, the sections were first incubated in mouse anti-rabies virus phosphoprotein (Raux et al., 1997), for 60 h at 4°C. Subsequently, they were incubated with Alexa®488-coupled donkey anti- mouse IgG (2 h, room temperature). The sections were then rinsed and mounted on glass slides using a 2% solution of gelatin in 0.05 M Tris/HCl (pH 7.6), dried at room temperature, dehydrated in toluene and coverslipped with Entellan. The sections were examined using a Zeiss 510 Meta confocal laser-scanning microscope (CLSM) with the appropriate band-pass and long-pass filter settings. A series of captured tiled CLSM images extending from the level of the prefrontal cortices to a mid optic chiasm level was analyzed for each case. The number of rabies virus-labeled neurons in each cortical layer and in each cortical area were reconstructed and counted. Layer 5 was divided into an upper and lower half in the case of primary cingulate cortex (Cg1), primary motor cortex (M1), secondary motor cortex (M2), primary somatosensory cortex (SS1), secondary somatosensory cortex (SS2), primary and secondary auditory cortex (Aud), and granular insular cortex (GI). Distinct layers could not be identified for secondary cingulate cortex (Cg2) and the various midline prefrontal cortices (PFC). Thus, counts for these regions are not broken down by layer. The SNr injections labeled striatal neurons predominantly in rostral dorsal striatum. The cortical areas on which we focused our attention are those that are the major sources of input to rostral dorsal striatum (Veening et al., 1980; McGeorge and Faull, 1989; Glynn and Ahmad, 2002; Seger, 2013).

## Results

### EM Studies in Rats of Corticostriatal Terminals on SPNs

Consistent with our prior studies (Lei et al., 2004), we found that the spines of both presumptive dSPNs (D1+) and presumptive iSPNs (D1−) receive IT-type and PT-type inputs, but the majority of IT-type terminals synapsed on presumptive dSPN spines (D1+) and the majority of PT-type terminals synapsed on presumptive iSPN spines (D1−) (Table 1). The mean size of all axospinous IT-type terminals, irrespective of whether the target was a D1+ spine or a D1− spine, was found to be 0.546 µm, similar to what we reported previously from single-label EM studies of IT-type terminals (0.524 µm) (Reiner et al., 2010). As in our prior studies, PT-type terminals were substantially larger than IT-type terminals, with their mean size being 0.823 µm for all D1+ or D1− spines combined, which is slightly smaller than we reported previously from single-label EM studies of PT-type terminals (0.909 µm) (Reiner et al., 2010).

**Table 1:** **Size and targets of corticostriatal terminal types**.

Category	D1+ Spines	D1− Spines	All Spines	D1+ Dendrites	D1− dendrites	All Dendrites
Size of BDA+ IT Terminals on	***0.504μ ± 0.02 #***	***0.595μ ± 0.03 #***	0.546 μm ± 0.02*	0.627 μm ± 0.10	0.489 μm ± 0.05	0.558 μm ± 0.06*
Size of BDA+ PT Terminals on	0.839 μm ± 0.06	0.815 μm ± 0.03	0.823 μm ± 0.03*	0.911 μm ± 0.17	0.780 μm ± 0.07	0.864 μm ± 0.11*
% of BDA+ IT Terminals on	50.4%	42.6%	93.0%	3.5%	3.5%	7.0%
% of BDA+ PT Terminals on	***29.4% #***	***59.8% #***	89.2%	***6.9% #***	***3.9% #***	10.8%

Tabulation of the size and frequencies with which IT-type and PT-type terminals make synaptic contacts on D1+ and D1− spines and dendrites, as determined from EM analysis of tissue in which either IT-type or PT-type terminals were selectively labeled with BDA, and D1 spines and dendrites were immunolabeled. The first row shows the mean IT-type synaptic terminal size (±SEM) on D1+ and D1−negative spines and dendrites, the second row shows the mean PT-type synaptic terminal size (±SEM) on D1+ and D1−negative spines and dendrites, the third row shows the IT-type synaptic terminal targets as a percent of all IT-type terminals detected, and the fourth row shows the PT-type synaptic terminal targets as a percent of all PT-type terminals detected. *significant difference between IT-type and PT-type for bolded pair of target structures. # significant difference between bold and italicized pair of target structures.

**Figure 1:**
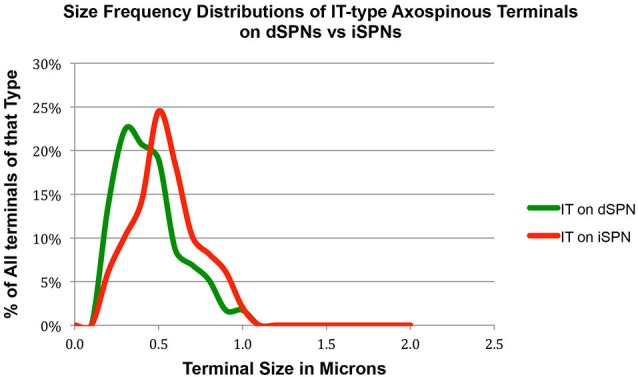
**Graph showing the size frequency distribution for IT-type input (as selectively visualized with BDA10k tracer labeling) to dSPN vs. iSPN spines, as determined from tissue that was immunolabeled for D1 to distinguish spine types**. Note that IT-type terminals on iSPN spines are larger than those of dSPN spines. For this graph, the abundance of terminals of each type is plotted per 0.1 µm increment in size, using Excel.

Our analysis here, however, revealed important new features of these corticostriatal inputs, for both spines and dendrites. For example, we observed a significant difference in the mean size of IT-type corticostriatal axospinous endings between presumptive dSPNs and iSPNs (*p* = 0.0165). In particular, although the size of PT-type terminals on D1+ spines was indistinguishable from that on D1− spines, IT-type terminals on D1− presumptive iSPN spines were on average significantly larger than those on D1+ presumptive dSPN spines (Figure 1; Table 1). Consistent with this, the size frequency distributions for IT-type axospinous endings on D1+ presumptive dSPNs differed from that for D1− presumptive iSPNs, with a peak at 0.3−0.4 µm for those on D1+ spines and a peak of 0.5 for those on D1− spines (Figure 1). By contrast, the size frequency distributions of PT-type axospinous endings did not differ notably between D1+ presumptive dSPNs and D1− presumptive iSPNs (not shown).

**Figure 2:**
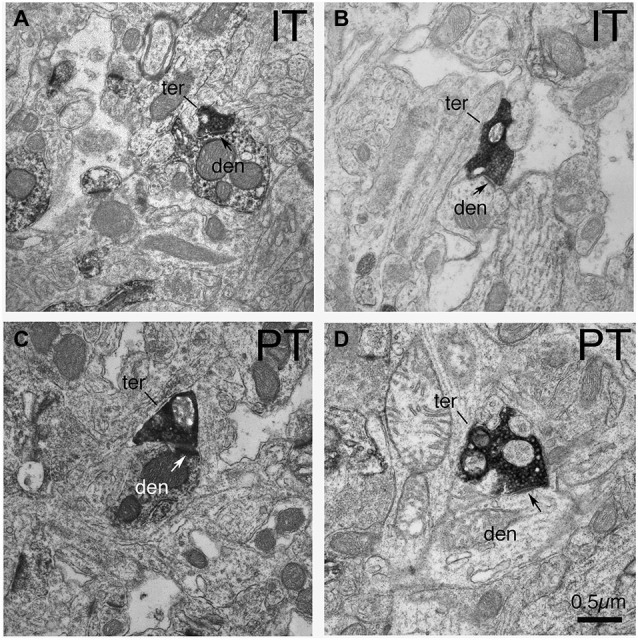
**EM micrographs showing IT–type and PT-type axodendritic synaptic terminals (as selectively visualized with BDA tracer labeling) in tissue that was immunolabeled for D1**. **(A)** IT-type synaptic terminal on D1+ dendrite. **(B)** IT-type synaptic terminal on D1-negative dendrite. **(C)** PT-type synaptic terminal on D1+ dendrite. **(D)** PT-type terminal on D1-negative dendrite. Note that PT-type synaptic terminals (ter) on dSPN (D1+) dendrites (den) are larger than those on iSPN dendrites (D1−negative). Postsynaptic densities are indicated by arrows. The magnification is the same in all images.

In the case of axodendritic endings, we found that IT-type axodendritic synaptic terminals (0.558 µm) overall were significantly smaller (*p* = 0.042) than PT-type axodendritic synaptic terminals (0.864 µm), consistent with the size difference for these terminal types on spines (Figure 2; Table 1). We also found that a greater percentage of the PT-type terminals ending on D1+ structures synapsed upon dendrites than is the case for D1− structures (Table 1). For example, 18.9% of PT-type contacts on D1+ structures ended on dendrites, while only 6.2% of the PT-type contacts on D1− structures ended on dendrites, which is significantly different by a chi-square test (*p* = 0.0112). IT-type terminals were, by contrast, equally common on D1+ and D1− dendrites (Table 1). For the reasons discussed in the Methods section, the D1+ dendrites are highly likely to largely or exclusively represent dSPN dendrites, while the D1− dendrites are likely to represent the dendrites of iSPNs and parvalbuminergic interneurons, as well as perhaps the rare calretinergic interneurons. Thus, PT-type synaptic terminals are more common on dSPN dendrites than iSPN dendrites, while IT-type terminals are apparently relatively equally common on dSPN and iSPN dendrites.

**Figure 3:**
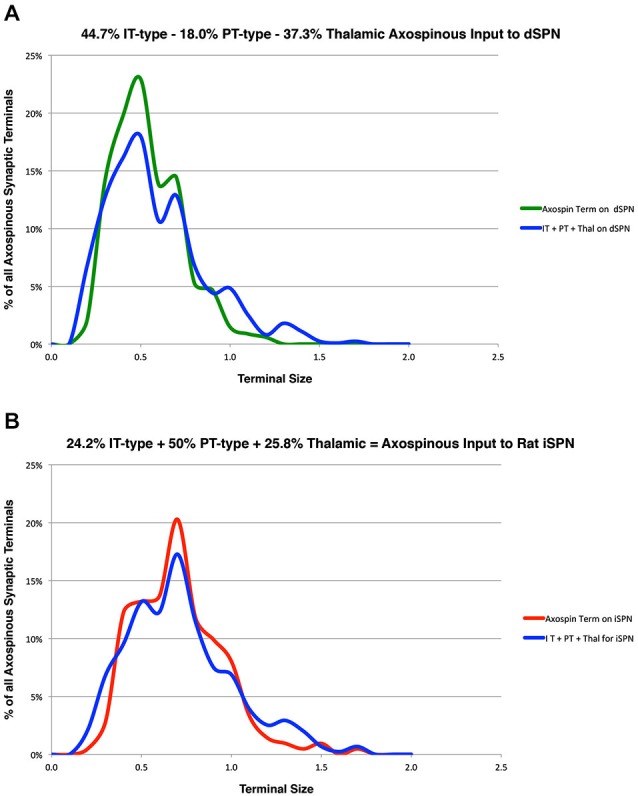
**Graphs showing: (A)** the proportions of IT-type, PT-type, and thalamic input that best account for the size frequency distribution of axospinous terminals on dSPNs reported previously by us (Reiner et al., 2010); and **(B)** the proportions of IT-type, PT-type, and thalamic input that best account for the size frequency distribution of axospinous terminals on iSPNs reported previously by us (Reiner et al., 2010). This analysis takes into account the differential size frequency distributions of IT-type axospinous endings on dSPNs vs. iSPNs found here, and the differential size frequency distributions of axospinous thalamic input to dSPNs vs. iSPNs reported in Lei et al. (2013). The size frequency distribution of PT-type axospinous terminals was as in Reiner et al. (2010) and Lei et al. (2013). The go-pathway dSPN size frequency distribution is color-coded green in **(A)**, while the stop-pathway iSPN size frequency distribution is color-coded red in **(B)**. For this graph, the abundance of terminals of each type is plotted per 0.1 µm increment in size, using Excel.

In prior studies, we used curve fitting to estimate the relative proportions of the spines of the two SPN types receiving IT-type, PT-type and thalamic synaptic terminals (Reiner et al., 2010; Lei et al., 2013). In our approach, we used our empirically determined size frequency distribution for each of these axospinous terminal types, and ascertained the relative abundance of each terminal type that summed to yield the best match to the known size frequency distribution of axospinous synaptic terminals on dSPNs vs. iSPNs. The size frequency distribution of axospinous synaptic terminals on dSPNs vs. iSPNs had been separately determined from our EM studies of retrogradely labeled neurons of these types (Reiner et al., 2010), to (Reiner et al., 2010; Lei et al., 2013). In Reiner et al. (2010), we had noted that the size frequency distribution for IT-type axospinous terminals closely matched that for dSPNs, while the size frequency distribution for PT-type terminals did not as closely match that for axospinous contacts on iSPNs. We took this to reinforce the conclusions that IT-type terminals preferred dSPN spines and PT-type terminals preferred iSPN spines. In Lei et al. (2013), we found slight differences between dSPNs and iSPNs in the size frequency distribution of their axospinous thalamic input. This allowed us to estimate the relative abundances of IT-type, PT-type and thalamic axospinous input to determine the best combination of each to match the size frequency distribution of axospinous synaptic terminals on dSPNs and iSPNs. Our results at that time suggested little to no PT-type input to dSPNs spines and relatively little IT-type input to iSPN spines. Our new findings on the relative sizes of IT-type axospinous endings on dSPNs and iSPNs are helpful in refining our curving fitting estimate of the relative abundances of IT-type, PT-type and thalamic axospinous inputs to dSPNs vs. iSPNs. Based on a best-fit curve-fitting approach, our present data indicate that a combination of 44.7% IT-type input 18.0% PT-type input 37.3% thalamic input best matches (at a 0.971 correlation) our measured size frequency distribution for the axospinous terminals on dSPNs (as determined for dSPNs detected by BDA3k retrograde labeling from nigra, Reiner et al., 2010; Figure 3). For iSPNs, our data indicate that a combination of 24.2% IT-type input 50.0% PT-type input 25.8% thalamic input best matches (at a 0.973 correlation) our measured size frequency distribution for the axospinous terminals on iSPNs (as determined for iSPNs detected by BDA3k retrograde labeling from GPe, Reiner et al., 2010; Figure 3).

**Figure 4:**
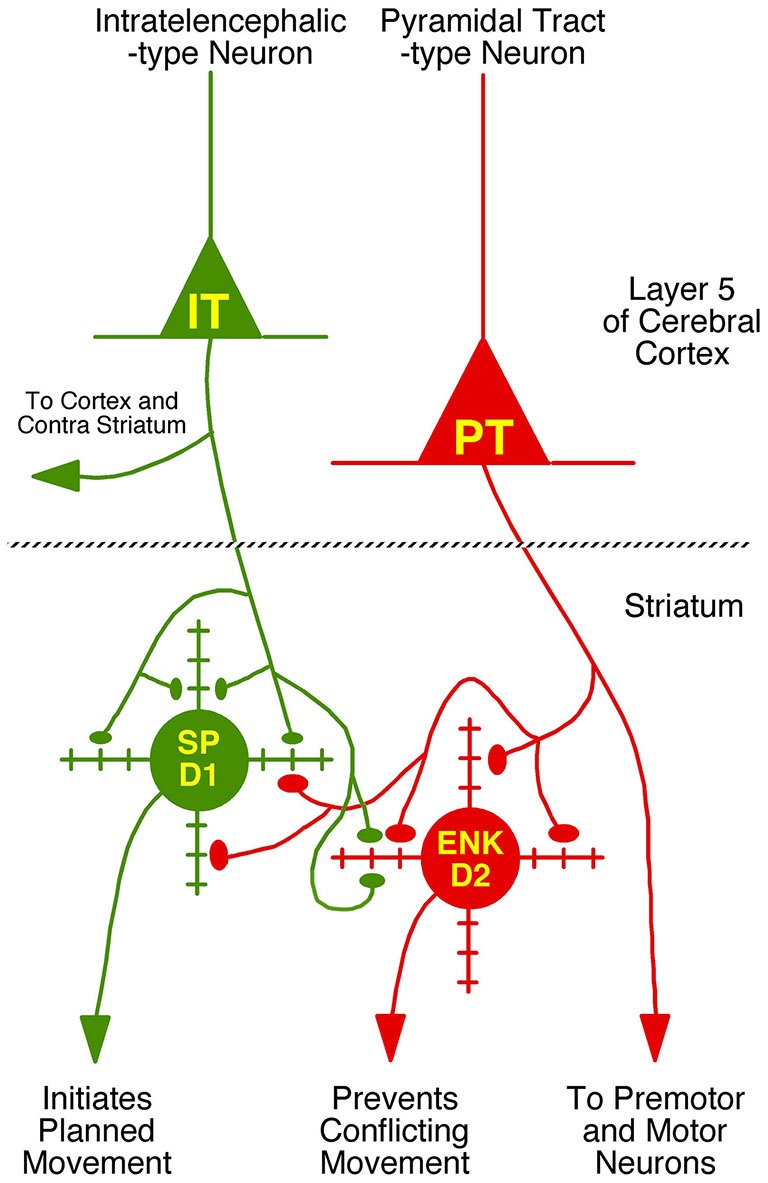
**Schematic illustration of the relative abundances of axospinous IT-type and PT-type inputs to dSPNs and iSPNs, based on the current composite findings shown in Table 2**. Both dSPNs and iSPNs receive axospinous IT-type and PT-type inputs, with dSPNs receiving about twice as much IT-type and iSPNs receiving twice as much PT-type axospinous input. Note that, as depicted, PT-type axospinous terminals are uniformly larger than IT-type axospinous terminals, and IT-type axospinous terminals on iSPNs are slightly larger than IT-type axospinous terminals on dSPNs. Axodendritic IT-type and PT-type terminals on dSPNs and iSPNs are far less numerous than axospinous terminals, with axodendritic representing 7% of all IT-type terminals to SPNs and about 11% of all PT-type terminals to SPNs. Notably, while axodendritic IT-type terminals are equally common on dSPNs and iSPNs, axodendritic PT-type terminals are about twice as abundant on dSPNs as on iSPNs. The schematic was created using Canvas and Adobe Photoshop.

We compared these estimates of the percent of spines on dSPNs or iSPNs receiving IT-type or PT-type input to counts of these types of synaptic contacts observed in material double-labeled for D1 and either IT-type or PT-type terminals. In the fields captured, we found that 24.6% of D1+ spines received IT-type synaptic terminals and 17.4% of D1+ spines received PT-type synaptic terminals. For D1−negative spines, we found that 24.1% received IT-type synaptic terminals and 28.1% received PT-type synaptic terminals in the fields captured. Since not all IT-type or PT-type terminals were labeled in these fields of view, we proportionally adjusted the percentages observed in the double-label material to total to the abundance of nonthalamic axospinous input for dSPNs and iSPNs reported in Lei et al. (2013). As shown in Table 2, we found that the abundance of IT-type terminals on dSPN spines was slightly less and the abundance of PT-type terminals on dSPN spines slightly more than estimated by the above-noted curve fitting. Similarly, the abundance of IT-type terminals on iSPN spines was relatively slightly more and the abundance of PT-type terminals on iSPN spines slightly less than estimated by the above-noted curve fitting. Why this might be is considered in the Discussion. In either case, our data indicate preferential but not exclusive IT-type input to dSPNs and preferential but not exclusive PT-type input to iSPNs (Figure 4). Averaging the two approaches, our results suggest that individual dSPNs receive about twice as many IT-type axospinous synaptic terminals as PT-type, and individual iSPNs receive about 1.5 times as many PT-type as IT-type axospinous synaptic terminals.

**Table 2:** **Abundance of corticostriatal terminal types on the spines of SPN Types**.

Category	% of spines with IT-type	% of spines with PT-type
**D1+ spines defined by IHC**	36.7%	26.0%
**D1− spines defined by IHC**	34.3%	39.9%
**dSPN spines by curve fitting**	44.7%	18.0%
**iSPN spines by curve fitting**	24.2%	50.0%
**Mean for dSPN spines**	40.7%	22.0%
**Mean for iSPN spines**	29.3%	45.0%

*Tabulation of the percent of D1+ and D1− spines receiving IT-type and PT-type axospinous synaptic contacts. The first two rows show the percentages for D1+ (dSPN) and D1−negative (iSPN) spines, respectively, as determined from EM analysis of tissue in which either IT-type or PT-type terminals were selectively labeled with BDA, and D1 spines were immunolabeled. The second two rows show the percentage IT-type and PT-type axospinous inputs for dSPN and iSPN spines, as determined by curve fitting as described in the text. The last two rows show the mean percent of dSPN and iSPN spines receiving IT-type and PT-type axospinous synaptic contacts based on these two approaches. As shown, both dSPNs and iSPNs receive axospinous IT-type and PT-type inputs, with dSPNs receiving more IT-type and iSPNs receiving more PT-type axospinous input*.

### Rabies Virus Retrograde Labeling in Rats

**Figure 5:**
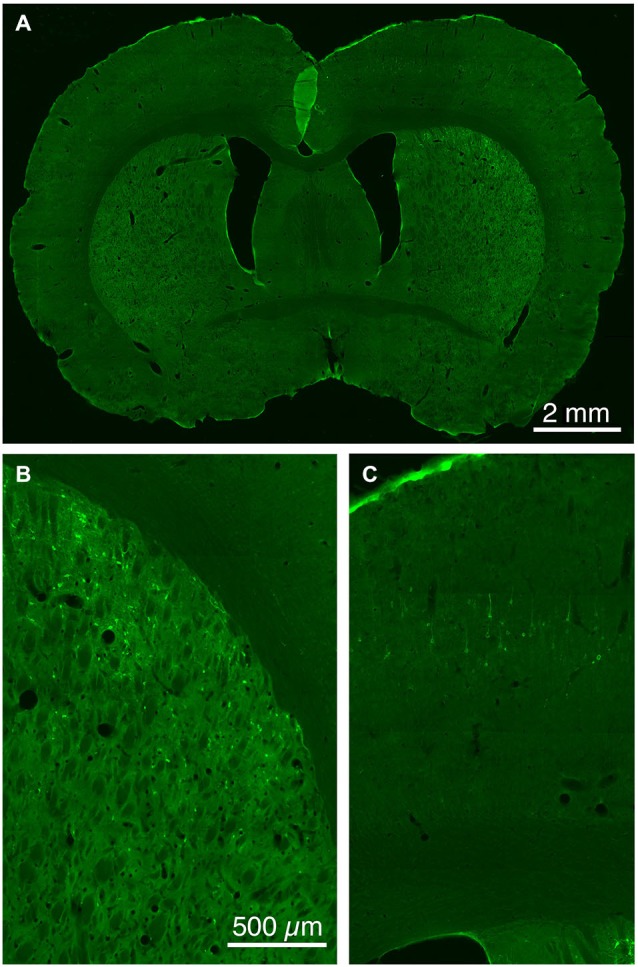
**Low (A) and higher power CLSM images of retrograde labeling of striatonigral dSPNs with rabies virus from the SNr (B), and transneuronal retrograde labeling of cortical neurons with rabies virus from the labeled striatonigral dSPNs (C)**. Magnification in **(B,C)** is the same.

**Figure 6:**
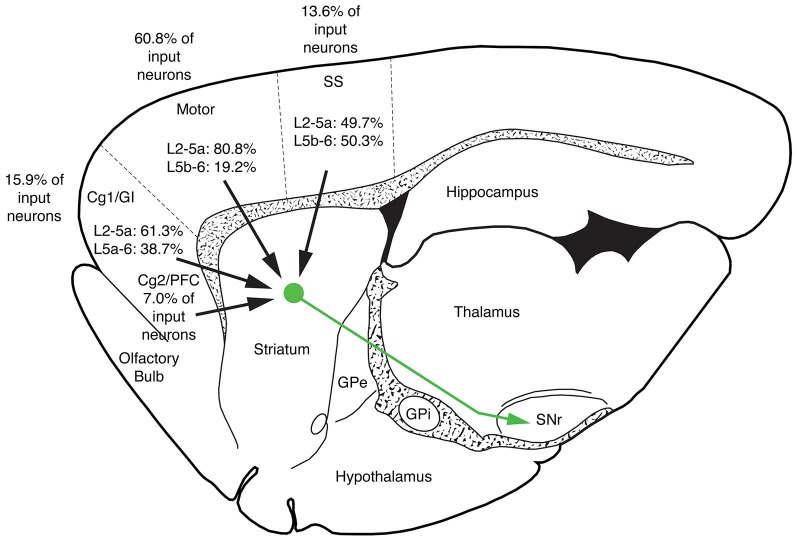
**Schematic illustration showing the relative abundance of cortical neurons selectively innervating dSPNs projecting to SNr in limbic cortices (cingulate, prefrontal and insular), M1/M2, S1/S2 and auditory cortex**. M1/M2 input predominates for striatonigral dSPNs. Note that no laminar breakdown is shown for the secondary cingulate cortex (Cg2) and prefrontal cortex (PFC) because distinct layers are not evident for these cortices.

The five cases analyzed involved rabies virus injection in substantia nigra pars reticulata that yielded labeled neurons in rostral dorsal striatum. In one case the labeled neurons were centered in dorsomedial striatum, in two they were centered in dorsal striatum, and in the final two they were centered in dorsolateral striatum. We found that the cortical neurons labeled in these cases and likely to largely represent the corticostriatal neurons projecting to these dSPNs (Figure 5; Table 3) mainly reside in the primary and secondary motor cortices (M1/M2) (60.8% of the labeled neurons for both sides combined) and primary and secondary somatosensory cortices (S1/S2) (13.6% of the labeled neurons for both sides combined), with the contralateral abundance being 12.1% of the overall total number of neurons labeled (Table 3). By contrast, the secondary cingulate cortex (Cg2) and prefrontal cortex (PFC) contained only 7% of all labeled cortical neurons, and the primary cingulate cortex (Cg1) and granular insular cortex contained 15.9% of all labeled cortical neurons. In contralateral cortex, labeled neurons in upper layer 5 of M1/M2 were in a 3:1 ratio with labeled neurons in lower layer 5, and in a 5:3 ratio in the case of S1/S2 (Table 3). Since the labeled corticostriatal neurons in contralateral cortex are all IT-type, this result suggests that the neurons of origin for the contralateral IT-type input are mainly but not entirely in upper layer 5. For ipsilateral M1/M2, the upper to lower layer 5 ratio is the same as for contralateral M1/M2 (3:1), but the upper layer 5 to lower layer 5 ratio for ipsilateral S1/S2 is 1:1. This suggests that dSPNs receive mainly IT-type input from M1/M2 ipsilaterally, since the upper to lower layer 5 ratio of labeled neurons ipsilaterally matches the upper to lower layer 5 ratio that the contralateral labeling shows typifies the laminar distribution of IT-type neurons. Using similar reasoning, the greater proportion of lower layer 5 neurons ipsilaterally than contralaterally suggests ipsilateral S1/S2 input to dSPNs includes input arising from PT-type neurons, as well as from IT-type neurons. Lower layer 5 input to dSPNs is also prominent for the limbic cortices (Table 3). The overall results suggest that dSPNs of the matrix compartment projecting to SNr do receive input from more IT-type than PT-type cortical neurons, especially from motor cortex (Figure 6).

**Table 3:** **Regional and laminar abundance of cortical neurons innervating dSPNs projecting to SNr**.

Cortical Region	Cg2/PFC	Cg1/GI	M1/M2	S1/S2	Auditory	Total
Side of Brain	% of All Labeled Neurons	% of All Labeled Neurons	% of All Labeled Neurons	% of All Labeled Neurons	% of All Labeled Neurons	% of All Labeled Neurons
**Contralateral**	1.12%	2.25%	7.49%	0.98%	0.23%	12.07%
**Ipsilateral**	5.83%	13.66%	53.33%	12.60%	2.52%	87.93%
**Combined**	6.95%	15.91%	60.82%	13.58%	2.75%	100.0%
**Contralateral**	**Laminar**	**Laminar**	**Laminar**	**Laminar**	**Laminar**	**Laminar**
	**Abundance**	**Abundance**	**Abundance**	**Abundance**	**Abundance**	**Abundance**
**Layer 2/3**	N/A	4.7%	11.0%	0.0%	0.0%	8.5%
**Layer 5a**	N/A	32.1%	67.7%	54.3%	54.5%	58.9%
**Layer 5b**	N/A	50.9%	19.8%	34.8%	36.4%	27.9%
**Layer 6**	N/A	12.3%	1.4%	10.9%	9.1%	4.7%
**Ipsilateral**	**Laminar**	**Laminar**	**Laminar**	**Laminar**	**Laminar**	**Laminar**
	**Abundance**	**Abundance**	**Abundance**	**Abundance**	**Abundance**	**Abundance**
**Layer 2/3**	N/A	8.4%	17.9%	1.9%	7.6%	13.6%
**Layer 5a**	N/A	57.0%	63.2%	47.5%	29.4%	58.7%
**Layer 5b**	N/A	31.7%	16.8%	45.3%	29.4%	24.0%
**Layer 6**	N/A	3.0%	2.1%	5.4%	33.6%	3.7%

*Tabulation of the regional abundance and laminar distribution of cortical neurons labeled transneuronally with rabies virus from striato-SNr dSPNs. The first three rows show the percent distribution of the labeled neurons in different areas of contralateral cerebral cortex, ipsilateral cerebral cortex, and all of cerebral cortex, expressed as a percent of all labeled cortical neurons in each case. Note that M1/M2 contains more labeled neurons than any other region on each side of the brain. The remaining rows show the laminar distribution of labeled neurons for each of the same regions on both sides of the brain. In this case, the frequency for each Layer is expressed as the percent of the total in that region. Note that upper Layer 5 input predominates for motor cortex on both sides of the brain*.

## Discussion

### Axospinous Terminals on Striatal Projection Neurons

Cortical input to striatum arises from two neuron types: (1) IT-type layer neurons that are involved in pre-movement planning; and (2) PT-type layer neurons that transmit motor commands to hindbrain and spinal cord (Turner and DeLong, 2000; Beloozerova et al., 2003; Reiner et al., 2010). The perikarya of IT-type neurons are mainly but not exclusively found in layer 5a, and the perikarya of the PT-type neurons are mainly but not exclusively found in layer 5b. Our present findings are consistent with our prior studies in showing that IT-type input favors dSPNs and PT-type input favors iSPNs. Our new data showing a size difference between IT-type axospinous endings on dSPNs and those on iSPNs has allowed us to refine our curve fitting estimates of the cortical inputs to the spines of dSPNs and iSPNs. This approach indicates that a combination of 42.7% IT-type input 18.0% PT-type input 37.3% thalamic input best matches our measured size frequency distribution for the axospinous terminals on dSPNs (as determined for dSPNs detected by BDA3k retrograde labeling from SNr). Conversely, our data indicate that a combination of 24.2% IT-type input 50.0% PT-type input 25.8% thalamic input best matches our measured size frequency distribution for the axospinous terminals on iSPNs (as determined for iSPNs detected by BDA3k retrograde labeling from GPe). The 3:1 predicted preference of PT-type axospinous terminals for iSPN spines (50.0%:18.0%) compared to dSPN spines indicated by this curve fitting is largely consistent with the results of the present EM double-label analysis, in which we observed that 67% of PT-type axospinous synaptic terminals end on iSPN spines and 33% end on dSPN spines (67%:33% ~2:1). The predicted 2:1 preference of IT-type terminals for dSPN compared to iSPN spines (47.7%:24.2%) indicated by this curve fitting is also largely consistent with our current EM double-label analysis, in which we observed that 54% of IT-type synaptic terminals in striatum end on dSPN spines and 46% end on iSPN spines.

In our EM double-label studies, however, we did observe more PT-type terminals on D1+ spines and more IT-type terminals on D1−negative spines than predicted from our curve fitting. It is uncertain whether this reflects some imprecision in the use of D1− immunolabeling as a means for D2 spine detection in our double-label EM studies, and/or some bias in the part of the dendritic tree whose spines are detected by D1 immunolabeling. There also could be some bias in the part of the dendritic tree whose spines are detected by retrograde labeling of SPN types (for example a bias toward proximal dendrites and their spines), and thus in the SPN axospinous size frequency distributions to which we are attempting to fit. It also may be that further refinement of the size frequency distributions of IT-type, PT-type, or thalamic terminals on dSPNs and iSPNs is needed. Finally, the slight mismatch between our curve-fitting and double-label EM studies for dSPNs could also stem from the existence of IT-sized terminals on dSPN spines from the small input to dorsolateral striatum from hippocampus, amygdala or posterior cortices (e.g., visual) (Veening et al., 1980; McGeorge and Faull, 1989; Kita and Kitai, 1990; Glynn and Ahmad, 2002; Seger, 2013). Similarly, the slight mismatch between our curve-fitting and double-label EM studies for iSPNs could stem from the existence of PT-sized terminals on iSPN spines from the small input to dorsolateral striatum from hippocampus, amygdala or posterior cortices. Studies with reporter mice in which all or nearly all IT-type terminals, or all or nearly all PT-type terminals are labeled, as now possible using IT-specific and PT-specific mouse cre lines (Gerfen et al., 2013), may allow more detailed characterization of the abundance and distribution of these terminal types along the extent of the dendritic trees of individually labeled dSPN and iSPNs. This approach will also make it more readily possible to assess if the striatum is regionally uniform in the organization of the IT-type and PT-type inputs to the SPN types. It is possible, however, that this approach will have the difficulty that mouse corticostriatal organization differs from that in other mammalian species. For example, cortical and thalamic axodendritic synaptic contacts on SPNs are considerably more abundant in rats and monkeys than they are in mice (Doig et al., 2010; Deng et al., 2013), and thus the allocations of IT-type and PT-type endings to SPNs may differ between mice vs. rats and monkeys as well. Nonetheless, we have found that, like in rats and monkeys, axospinous terminals on iSPNs in mice are larger than those on dSPNs (Reiner et al., 2010; Deng et al., 2014), suggesting that dSPNs and iSPNs in mice too may differ in the relative proportions of IT-type and PT-type axospinous inputs they receive.

### Axodendritic Terminals on Striatal Projection Neurons

Our present EM double-label findings also suggest that dSPNs receive a more substantial axodendritic PT-type input than do iSPNs. The difference may be greater than the nearly twofold difference we saw using D1 immunolabeling to detect these dendrite types, since some of the PT-targeted D1− dendrites may have belonged parvalbuminergic or calretinergic interneurons, as noted in the Methods section. Moreover, the size of PT-type axodendritic synaptic terminals on dSPNs also appears to be somewhat greater than those on iSPNs. Thus, it might be expected that dSPNs would respond to activation of PT-type terminals, given they receive both axospinous and prominent axodendritic PT-type input. The magnitude of this activation would depend, in part, on the relative efficacies of the axospinous vs. axodendritic PT-type inputs. By contrast to PT-type terminals, IT-type terminals make far fewer synaptic contacts with D1+ and D1− striatal dendrites, and they appear relatively equal in their targeting of them. Our confocal laser scanning microscope analysis of cortical inputs to striatum labeled by VGLUT1 immunolabeling shows that the cell bodies of SPNs are surrounded by VGLUT1+ terminals (Deng et al., 2013; Lei et al., 2013). EM examination of these terminals reveals, however, that they invariably do not synapse on the perikarya that they abut. Rather, they typically synapse on nearby spines. Thus, axosomatic input to SPNs is meager, as reported previously by others (Frotscher et al., 1981; Somogyi et al., 1981), and unlikely to contribute notably to differences between SPN types in responses to IT-type and PT-type inputs.

### Functional Analysis of Differential IT-type and PT-type Inputs to SPNs

A number of electrophysiological studies support our findings on differential cortical input to SPNs (Uhl et al., 1988; Berretta et al., 1997; Parthasarathy and Graybiel, 1997; Mallet et al., 2006; Cepeda et al., 2008; Ding et al., 2008; Kreitzer and Malenka, 2008; Takada et al., 2013). These studies generally show that cortical activation is more effective in activating iSPNs than dSPNs, as might be expected given the larger size of PT-type than IT-type terminals (Sulzer and Pothos, 2000), and their preferential axospinous input to iSPNs. One recent study took a different approach and showed that immunotoxin-mediated depletion of PT-type neurons in monkeys reduces cortical activation of iSPNs but not dSPNs (Takada et al., 2013). On the other hand, another recent study using optogenetics (Kress et al., 2013) reported equal dSPN and iSPN responsiveness to IT activation, and greater dSPN than iSPN responsiveness to PT activation. The basis of the discordance between these results and those that might be expected from our morphological findings is uncertain. A greater responsiveness of dSPNs to PT-type input may have occurred, in part, because dSPNs receive more prominent dendritic PT input than do iSPNs, as the present findings now indicate. The equal responses of dSPNs and iSPNs to IT-type optogenetic activation may have occurred due to the sizable IT-type input to iSPNs, and the larger size of these terminals on iSPNs than dSPNs. Morita (2014) has also suggested, based on a computer modeling approach and published data on paired-pulse ratios for the cortical inputs to SPNs, that differential short-term plasticity for corticostriatal terminal types on dSPNs and iSPNs may account for the difference between Kress et al. (2013) and our findings. In particular, he has suggested that IT-type inputs to dSPNs and PT-type inputs to iSPNs may show short-term facilitation whereas IT-type inputs to iSPNs and PT-type inputs to dSPNs may show short-term depression (Morita, 2014). Thus, single pulse optogenetic activation would show large iSPN responses to IT activation and large dSPN responses to PT activation. Repetitive IT or PT activation would have larger effects on dSPNs and iSPNs, respectively, consistent with the preferential IT-type axospinous input to dSPNs and PT-type axospinous input to iSPNs that we have observed morphologically.

### Regional and Laminar Source of Cortical Inputs to SPNs

In the present study, we also injected CVS-11 rabies virus into rat SNr to identify the cortical neurons projecting to matrix striatonigral dSPNs. The post-injection survival time (40–42 h) was chosen to limit labeling to cortical neurons projecting directly to striato-SNr neurons, and limit cortico-cortical or thalamo-cortical routes of labeling. We found that corticostriatal neurons projecting to dSPNs of dorsal striatum appear to mainly reside in M1/M2 (60.8%) and S1/S2 (13.6%), with the contralateral neuron abundance being about 12% of the ipsilateral abundance. Our results also indicate that dSPNs projecting to SNr (which reside in the matrix compartment) receive mainly IT-type input from M1/M2 ipsilaterally and contralaterally, based on the laminar and regional location of the corticostriatal neurons projecting to them (Reiner et al., 2003). Our data also suggest that the sources of input to dSPNs from the somatosensory cortices includes both IT-type and PT-type neurons. Thus, S1/S2 at least is seemingly a major source of PT-type input to dSPNs. Since IT-type perikarya can reside in layer 5b and PT-type perikarya can reside in layer 5a (Reiner et al., 2003), however, input of PT-type neurons of the motor cortices to dSPNs cannot be ruled out merely on the basis of the location of the labeled neurons. The preponderance of layer 2/3 and 5a neurons among those labeled with rabies virus suggests, in any event, that dSPNs do preferentially receive input from IT-type cortical neurons. This observation further argues against a significant contribution to cortical labeling via retrograde labeling of GPe or STN from the SNr injections with RV. Had that been the case, many more deep layer 5 neurons would have been labeled, since they are the source of the cortical projections to GPe and STN (Inoue et al., 2012). By their nature, however, the data cannot be used to reach conclusions about the relative abundance of axospinous vs. axodendritic IT-type synaptic terminals vs. PT-type synaptic terminals on dSPNs from any given regional or laminar source, since each neuron retrogradely labeled cannot be assumed to give rise to an equal number of terminals. It should be noted, however, that we observed that rabies virus-labeled neurons in upper vs. lower layer 5 are about as abundant as expected based on the approximately 2:1 ratio for IT:PT input to dSPNs suggested by the present EM work in rats. By contrast, Wall et al. (2013) used pseudotyped monosynaptic rabies virus to label cortical inputs to dSPNs in mice, and found that the ratio of virus-labeled neurons in upper vs. lower layer 5 was about 5:1, rather than the 2:1 expected based on the present EM work in rats. The basis of this is uncertain. It may be that the highly convergent nature of the IT-type input and the more discrete nature of the PT-type input (Wilson, 1987; Cowan and Wilson, 1994; Wright et al., 1999, 2001; Reiner et al., 2010) contributes to this difference. If so, however, then this factor is not as prominent in rats. It also may be that the laminar distribution of IT-type and PT-type neurons differs between rats and mice, or that the virus used by Wall et al. (2013) is for some reason more preferentially taken up by IT-type terminals.

Our results with rabies virus transneuronal retrograde labeling from SNr in rats also differ from those of Wall et al. in terms of the cortical regions projecting to dSPNs. They reported that somatosensory and limbic cortical structures preferentially innervated dSPNs, whereas motor cortex preferentially targets iSPNs. By contrast, we found that the primary and secondary motor cortex contained the majority of neurons innervating dSPNs projecting to SNr in rats. A number of factors may account for this difference. First, our corticostriatal labeling was confined to neurons projecting to striato-nigral dSPNs projecting to SNr. By contrast, the strategy that Wall et al. used would have yielded labeling of cortical neurons with input to both striato-GPi and striato-nigral dSPNs, as well as to striosomal SPNs expressing D1. Inclusion of striosomal SPNs among the targets of cortical input, in particular, may explain why limbic regions were more prominent among those showing retrograde labeling in Wall et al. than in our own study, since limbic cortices project heavily to striosomes (Gerfen, 1989; Kincaid and Wilson, 1996).

Given these considerations, the recent results of Spigolon et al. (2013) are of interest. They used a pseudotyped monosynaptic rabies two-virus system to transfect cortical neurons projecting to either dSPNs or iSPNs with GFP or ChR2. They reported equal upper (presumptive IT-type) and lower layer 5 (presumptive PT-type) inputs to iSPNs from barrel somatosensory cortex in mice, in contrast to Wall et al. (2013) who reported preferential upper layer 5 (presumptive IT-type) input from somatosensory cortex to iSPNs. Input to dSPNs from barrel cortex was preferentially from upper layer 5 in Spigolon et al. (2013), as well as for somatosensory cortex in Wall et al. (2013). Spigolon et al. also noted that neurons projecting from somatosensory cortex to dSPNs were typically smaller and possessed finer dendrites than did those projecting to iSPNs, consistent with a preferential IT-type input to dSPNs and preferential PT-type input to iSPNS. Finally, Spigolon et al. (2013) reported that optogenetic stimulation of motor cortex layer V corticostriatal neurons that had been selectively labeled with ChR2 from dSPNs induced contralateral rotation. No effect was seen on locomotion after stimulation of ChR2+ corticostriatal neurons in layer V of motor cortex projecting to iSPNs. The study of Spigolon et al. thus supports the existence of anatomical and functional differences between corticostriatal neurons targeting dSPNs compared to iSPNs, with those targeting dSPNs but not iSPNs eliciting movement, consistent with a major input to dSPNs from motor cortex and with the role of dSPNs in movement initiation.

### Functional Implications for Motor Control and Plasticity

In the case of dSPNs, convergence of IT-type input from sensory and motor cortical areas regarding movement planning, body position and the environment may provide the coherent input required to activate individual dSPNs so that they facilitate movement (Wilson, 1987; Cowan and Wilson, 1994). Thalamic input related to attention may provide further excitatory drive needed to push dSPN activation over the threshold for motor initiation (Smith et al., 2004, 2011). Our recent findings on corticostriatal pathology in Q140 Huntington’s disease heterozygous mice are consistent with this scenario (Deng et al., 2013, 2014). We found that dSPNs in Q140 mice experience a profound loss of their small corticostriatal axospinous synaptic terminals at 1 year of age, but iSPNs show no significant loss of corticostriatal terminals at this same age. Based on their small size, it seems likely that the terminals lost from dSPNs are IT-type. Regression analysis indicated that the loss of corticostriatal terminals to dSPNs was associated with mild hypokinesia at 1 year of age. Such deficits would be expected with loss of motor planning IT-type input.

Our findings suggest that iSPNs receive an efference copy of motor commands via their PT-type input, perhaps to suppress movements that would otherwise conflict with ongoing movements. The PT signal, however, will reach the brainstem and spinal cord before it reaches motor cortex via the iSPN-STN-GPi-motor thalamus loop, and thus be too late to prevent movements conflicting with the already initiated movement. Graybiel (2005) has suggested that PT-type input to iSPNs may serve to terminate a specific step in an action sequence initiated by dSPNs. In this regard, the PT-type input to dSPNs is also of interest, since the computational modeling considerations of Prescott et al. (2006) suggest that the maintenance of selected actions by the basal ganglia requires an efference copy signal for the selected action. The PT-type input to dSPNs may then serve to sustain actions beyond their initiation. Plasticity in this input might then play a role in modulating action duration to fit the task.

The PT feedback signal may also play a role in linking dSPN activity to movements that produce a desired outcome. In this scenario, the IT-type activation of a dSPN would be viewed as the presynaptic activation in a positive-timing spike timing dependent plasticity paradigm (pSTDP), while the PT-type input would represent the postsynaptic depolarization (Shen et al., 2008). In the presence of dopamine release acting on D1 receptors after a successful behavioral outcome, the temporal succession of IT-type and PT-type activation would, in essence, instruct a dSPN that IT-type activation had led to a movement with a desirable outcome, and facilitate those IT-type synapses, increasing the likelihood of their activation in that behavioral context. In the case of iSPNs, the temporal succession of IT-activation, PT-activation and dopamine reward acting on D2 receptors would, in essence, instruct a given iSPN that the IT-activation is associated with a desirable behavior, and the IT-activation of the iSPN neuron needs to be suppressed so that it does not cause the iSPN to inhibit the rewarded response. This may increase the precision of movement selection (Nishizawa et al., 2012). In a pSTDP experimental paradigm, however, the optimum delays between the presynaptic and the perikaryal depolarization are on the order of milliseconds (Kreitzer and Malenka, 2008; Pawlak and Kerr, 2008; Shen et al., 2008; Fino and Venance, 2010; Markram et al., 2012), while the delay between the IT-type activation and the PT-activation from the engendered movement will be an order of magnitude greater, even with the rapid conduction of PT-type axons (Cowan and Wilson, 1994). Despite such delays, the potentiation for dSPNs and depression for iSPNs may be sufficient to yield motor learning in the behaving animal.

## Conflict of Interest Statement

The authors declare that the research was conducted in the absence of any commercial or financial relationships that could be construed as a potential conflict of interest.
